# A preliminary analysis of the psychometric properties of the German O*NET interest profiler short form in rehabilitation education

**DOI:** 10.3389/fresc.2026.1789335

**Published:** 2026-08-20

**Authors:** Steffen Wild, Michélle Möhring

**Affiliations:** Rehabilitation Sciences, TU Dortmund University, Dortmund, Germany

**Keywords:** interest inventory, O*NET interest profiler, rehabilitation education, RIASEC, validity, vocational interests

## Abstract

Interest is a key driver of performances, decisions and choices, for example starting an educational program. The German O*NET Interest Profiler Short Form is a recent RIASEC-based instrument. However, its evidence is limited in German speaking subpopulation. We analyze its psychometric properties in *N* = 173 students from rehabilitation education programs. Internal consistency is adequate in all six scales (*ω* = .80–.90) and structural validity shows partial support for the RIASEC circumplex. Intercorrelations follow the expected pattern only partly and multidimensional scaling indicate a good overall fit (RSQ = .94; stress ≈.11), although deviations from a perfect hexagonal is observed. Discriminant validity is supported by generally small-to-medium associations with Big Five traits below the discriminant threshold (*r* < .85). Criterion-related validity is weak. Using Euclidean distance for congruence analysis, associations with study satisfaction (*r* = −.08, −.09) and dropout intention (*r* = .06) are near zero. Using angular agreement for congruence analysis, small but statistically significant correlations exist for study satisfaction (*r* = .17–.18, *p* < .05). In summary, results show partial evidence for structural validity, clear support for discriminant validity, and limited criterion-related validity. Findings further suggest that conclusions about validity depend on sample characteristics and the chosen operationalization of vocational interest congruence.

## Highlights

- We replicate the hexagon model structure from Holland's Inventory of interest (RIASEC) by multidimensional scaling with regard to the instrument's structural validity.
- The results provide initial evidence for the instrument's discriminant validity, based on the correlations between the vocational interest dimensions and the Big Five Inventory.
- The correlations between interest congruence and dropout intentions and study satisfaction show small and test dependent effect sizes, indicating weak evidence for a test dependent criterion validity of the instrument.
- We conducted our research based on a sample of students enrolled in the rehabilitation education study program, a population that has been understudied to date.

## 1 Introduction

In line with Holland's RIASEC theory (1), vocational interest is defined as individual interests with relatively stable preferences for activities, contexts, and outcomes (2–4). It is also a frequently addressed concept in various research areas, such as the labor market and personality (5). Empirical results indicate that interest is associated with job satisfaction, career and educational choices, performance, and turnover intentions (3, 6–8). In higher education, empirical results highlight the importance of students' interests for performance in their chosen study program (9). Interest fit between students and study programs is considered helpful for reflecting on study choices, but effects, e.g., with study program satisfaction, are smaller than in industry (10).

A key challenge lies in the measurement of interest. Most inventories have been developed and validated in the U.S. Therefore, there is a need for considering specific regional contexts in both the measurement itself and in higher education programs. In recent years, however, efforts have been made to address this gap (2, 11, 12). While Roemer et al. (2) make an important first validation of the German O*NET Interest Profiler Short Form on a specific sample among psychology students as well as high school students, evidence on its psychometric properties across different German-speaking subpopulations and applied contexts remains limited. Consequently, further studies that examine the instrument in specific educational and occupational contexts as well as settings are necessary to evaluate and contextualize the generalizability and boundary conditions of the initial findings.

Another challenge concerns Holland's calculus hypothesis, which has been criticized because the postulated hexagonal structure that geometrically represents the six vocational personality types as vertices positioned at 60-degree intervals for reflecting their psychological proximity, does not work consistently. Instead, a circumplex structure is discussed (13). Further debate concerns whether the correlation between interest and job satisfaction differs across different interest fits, debating a single-index fit that could described as a “one size fits all” measure (14).

With this study, we examine the validation of the German O*NET Interest Profiler Short Form (2), a new measurement instrument based on Holland's framework in the German language. The purpose of this research is to evaluate this instrument by examining different forms of validity. Structural validity is tested using content validity, internal consistency, item analyses and multidimensional scaling. Discriminant validity is assessed through correlations with the 5-factor model of personality, also known as Big Five Personality Traits (15), as there is a long tradition for comparing dimensions of Big 5 and RIASEC (16). Criterion validity is tested by analyzing the person-environment fit (congruence) in relation to students' study program satisfaction and dropout intentions. For this purpose, we use two different indices, the Euclidean distance and Angular agreement (17), to evaluate the robustness of the results. We conduct these analyses with data from students enrolled in the rehabilitation education program that does not lead to a clearly defined career path (e.g., compared to teaching). This population is understudied in higher education, and there is a shortage of skilled workers in the social sector (18, 19). Furthermore, rehabilitation education differs from highly standardized professional programs, like medicine, nursing, teacher education, or engineering, because it does not correspond to a single clearly bounded occupational environment. Instead, graduates enter heterogeneous work places, like educational, clinical, counselling, social, and rehabilitation-related contexts. Consequently, the environmental reference point required for Holland-type person–environment congruence is structurally diffuse in this population. This situation represents not merely a sample-specific limitation, but a theoretical boundary condition of congruence approaches, which assume relatively stable and clearly defined environmental profiles (1). In such contexts, weak congruence effects may therefore reflect limited environmental specificity rather than insufficient psychometric quality of the instrument itself. Current meta-analytic evidence already suggests that congruence effects in higher education are smaller and more heterogeneous than in occupational settings (10), possibly because study programs often provide less standardized environmental structures than occupations. Rehabilitation education may represent a particularly strong case of this fact due to its broad and multidirectional professional trajectories. The focus on a special student population is a deliberate methodological choice, as validation research commonly examines instruments within clearly defined groups to test context sensitivity and applicability. Especially, students in rehabilitation education represent a sophisticated group due to their heterogeneous career trajectories, wide specialized skill profiles and the comparatively low degree of occupational standardization. This allows stringent testing of interest structures and congruence analyses. The measurement instrument has not yet been applied to this group. We also chose this instrument, because it is freely available and needs to be tested across different academic professions and locations. Consequently, our study contributes to basic research, that helps to analyze academic success, as intrinsic academic motivation is associated with academic achievement (20–22).

This study uses a student sample from a specific salient domain and keeps the focus on key geometric assumptions. This procedure reflects our main aim to see whether theory-based relationships appear where interest structures are especially relevant. Thus, the focus on a specific subpopulation should not be understood as a limitation, but as a theoretically informed step toward cumulative validation across diverse groups. It is not intended to do a full test of Holland's calculus hypothesis across different populations.

In our study, we have two overarching goals. The first goal is to gain evidence for the validity of the instrument by linking it to the theoretical assumptions of the RIASEC model through specific analytical tests (e.g., structural relations, nomological network, and congruence outcomes). The secondary goal is to explore the distances between RIASEC dimensions to examine whether the expected geometric ordering, specifically the calculus hypothesis, is reflected in the data and how discrepancy influences the interpretation of congruence results.

## 2 Theoretical background

### 2.1 Holland's RIASEC model of interest

Holland's vocational interest framework is based on a classification system (1, 23), referred to as the RIASEC model. It proposes six domains that represent vocational interest types: Realistic (R), Investigative (I), Artistic (A), Social (S), Enterprising (E), and Conventional (C). Each domain is a constellation of interests covering activities, beliefs, abilities, values, and characteristics, and can be used to describe both an individual's interests, and a person's work environment supplies (1, 23, 24). A more detailed explanation postulates (1, 2, 23, 24) that Realistic (R) interests involve working physically with hands, objects or tools (e.g., woodworking; mechanics). Investigative (I) interests are involved in scholarly, research, or scientific activities and characterized as thinking through problems. Artistic (A) interests follow the idea of being creative and expressive (e.g., painting a portrait, dancing). Social (S) interests are reflected in activities and occupations that involve helping, nurturing, serving, or teaching (e.g., nursing; elementary school teaching). Enterprising (E) interests involve business-related or influencing activities, such as selling, managing, and leading. Finally, Conventional (C) interests integrate work that is structured and systematic (e.g., tracking shipping records, accounting).

The underlying calculus hypothesis is a further element of the RIASEC framework (1). According to this hypothesis, the six domains are arranged in a hexagon (or circle). This structure further assumes that adjacent interest domains (e.g., Realistic and Investigative) are more strongly related to each other than alternate domains (e.g., Realistic and Artistic), while opposite domains are the least related to each other (e.g., Realistic and Social) (2). However, it is currently being discussed whether the hexagonal structure can be maintained or whether a circumplex structure, polygonal, or elliptical structure would be more appropriate (25, 26). The mentioned structural representations—hexagonal, circumplex, polygonal, and elliptical—refer to different spatial models used to illustrate relationships among vocational interests. In our study, these terms are introduced only to acknowledge the ongoing debate about the geometry of interest models, not to analyze structural differences in depth. Therefore, a detailed explanation of each representation would exceed the focus and aims of the present work.

Another concept in Holland's framework is the congruence assumption. It is based on the general approach of the Person-Environment (P-E) Fit theory (6, 27). In this context, congruence refers to the alignment between a person's personal interest type and the characteristics of a professional environment, e.g., an artistic type in an artistic environment (24). Empirical results confirm that higher congruence is positively associated with job satisfaction and life satisfaction, negatively associated with turnover intentions (17, 28), and also linked to academic success, such as positively predicting academic achievement, persistence in the study program, and satisfaction with the study program (10). Nye et al. (29) conclude that congruence indices often predict job performance better than interest scores alone and even simple measures, such as matching first-letter codes of highest interest domains, can improve validity. In this way researchers discuss the model of polynomial regression. More indices like Euclidean distance, Angular agreement, Profile deviance or Profile correlation are discussed (17).

Vocational interests are embedded in a nomological network that links them to other individual differences concepts (2, 30). A long tradition exists between analyzing vocational interests and the Big Five personality traits (16), seen as one element in a nomological network. A meta-analysis by Mount et al. (31), reported four correlations (p) equal or greater than .20: Openness to Experience-Artistic (*ρ* = .41), Extraversion-Enterprising (*ρ* = .40), Extraversion-Social (*ρ* = .29), and Openness to Experience-Investigative (*ρ* = .25). A meta-analysis by Larson et al. (32) found similar correlations. Current research confirms these associations and provides further evidence for the relation between Agreeableness-Social [*r* = .32 by Roemer et al. (2); *r* = .25 by van der Linden et al. (16)].

Although the Big Five personality traits and vocational interests represent empirically distinct constructs, they show systematic and theoretically meaningful associations with small to medium effects. This association pattern supports the use of the Big Five as a relevant external criterion in studies analyzing discriminant validity within RIASEC research. Parallel, it highlights that vocational interests capture variance beyond broad personality dimensions.

### 2.2 The study program of rehabilitation education

In Germany and neighboring countries, rehabilitation education typically aims to enable people with chronic impairments or illnesses to cope with their disabilities and their consequences, as well as to participate in everyday life in society in a self-determined and largely independent manner, and, when necessary, through integrated intervention programs (33, 34). It can be assumed that students in rehabilitation education programs may contribute to these goals from an educational perspective for individuals of all ages. For this purpose, these students are not necessarily trained as special educational school teachers.

Rehabilitation education is considered an independent field in pedagogy (35, 36). Related disciplines are curative pedagogy, sociology, psychology, and medicine. There are further subsections, like inclusive education. Depending on the focus, a particular discipline is emphasized. This interdisciplinarity standing implies that rehabilitation education cannot easily be represented by a single vocational environment within Holland's framework. In contrast to occupationally standardized study programs, students may orient themselves toward substantially different future roles and work settings already during their time at university. Therefore, the environmental profile used in congruence analyses is likely to represent only a partial abstraction of the actual opportunity structure available to students. This equivocalness is theoretically highly important, because Holland's congruence model presupposes sufficiently coherent environmental profiles against which individual interests can be compared. Rehabilitation education has gained an international scientific standing, because dedicated journals such as Rehabilitation Education (37), have been established.

Students in rehabilitation education are characterized by their knowledge across a wide range of areas. For example, they acquire fundamental knowledge about lifelong learning (38). They develop skills for rehabilitation education decisions using diagnosis, promotion and rehabilitation planning, and rehabilitation-pedagogical intervention (39). Training in research methods is another component of this academic field (40).

Curricula of rehabilitation education programs include modules in rehabilitation sciences, pedagogy, psychology, assessment, vocational rehabilitation practice, and supervised internships, as shown in official study and examination plans (39, 40). This clarification is necessary, because it describe typical training content of students rather than full professional knowledge, which applies more to graduates. The students in rehabilitation education are a relevant group for our research questions about vocational interests because they are in a key position for vocational choice and transition into rehabilitation professions, that is covered by the O*NET RIASEC framework. Therefore, this section motivates why rehabilitation students are an appropriate sample for examining vocational interests within the O*NET RIASEC framework and leads directly to the research questions and hypotheses of this study.

### 2.3 The present study

We investigate the validity of the German O*NET Interest Profiler Short Form (2), an instrument for measuring vocational interests based on Holland's theoretical model (1), in a sample of university students enrolled in the rehabilitation education program. This research is necessary, because this population is understudied, and the German O*NET Interest Profiler Short Form has not been sufficiently tested psychometrically, for example in German-speaking populations. Consequently, the instrument needs to be evaluated for robustness of its psychometric test criteria. Most inventories for vocational interests are developed and validated in the U.S., and applying interest inventories across different cultures can pose various challenges (12, 41). Holland's calculus hypothesis has been criticized and should be evaluated, as the hexagonal structure does not work consistently (13). Further debate has arisen regarding the fact that the correlation between interest and job satisfaction varies across different congruence indices (14).

The strategy for the analyses is directly retrieved from the theoretical framework. This is done because the RIASEC model implies (a) a geometric structure [in this study analyzed by multidimensional scaling (MDS) and intercorrelations], (b) distinct but related constructs (checked through correlations with Big Five), and (c) meaningful outcomes of congruence (tested via relations to study satisfaction and dropout intentions). Consequently, we analyze the following objectives.

Accordingly, the overall research objective of the present study is to examine the structural, discriminant, and criterion validity of the German O*NET Interest Profiler Short Form in a sample of university students enrolled in rehabilitation education. To address this, the following research questions and hypotheses were derived:

Research question 1 (RQ 1): How much is the theoretical hexagonal structure of Holland's RIASEC model replicated in the German O*NET Interest Profiler Short Form within a sample of university students in rehabilitation education?
Research question 2 (RQ2): To what extent do the RIASEC dimensions demonstrate discriminant validity through their relations with the Big Five personality traits?
Hypothesis 1: It is hypothesized that the Big Five personality traits will correlate with the RIASEC dimensions, but that the correlations will remain below *r* < .85 (pointing to discriminant validity).
Research question 3 (RQ3): To what extent is person–environment congruence associated with study satisfaction and study dropout intention?
Hypothesis 2: It is hypothesized that student satisfaction will be positively associated with person–environment congruence, whereas study dropout intention will be negatively associated with congruence, with correlations expected to be statistically significant (*r* > |.10|).

## 3 Methods

### 3.1 Participants and design

Data for this study were collected as part of the non-funding project “Neigungen und Interessen von Studierenden der Rehabilitationspädagogik [Aptitudes and interests of students in rehabilitation education] (NISt-Re)”. Students were invited to participate voluntarily in the summer term of 2025. The final dataset includes *N* = 173 students (83 percent female; age *M* = 24.57; *SD* = 5.29). A cross-sectional design and convenience sampling were applied.

In this online survey, data were collected at four different universities, these are the universities for studying rehabilitation education in Germany. The study achieved a response rate of approximately 15%. Participants were enrolled in a bachelor's study program (*n* = 126; 73%) and a master's study program (*n* = 47; 27%). See Table 1 in the Supplementary Material for detailed participant demographics.

**Table 1:** Psychometric characteristics of scales.

Construct	Scale	*k*	*ω*	*p_i_*	*r_it_*	Item sample	Source
Vocational Interests						Please indicate how interested you are in these activities.	(2)
	Realistic	10	.87	.09–.31	.48–.73	Lay brick or tile.	
	Investigative	10	.90	.21–.44	.34–.70	Develop a new medicine.	
	Artistic	10	.88	.48–.54	.48–.71	Compose or arrange music.	
	Social	10	.80	.47–.81	.39–.61	Help conduct a group therapy session.	
	Enterprising	10	.84	.17–.46	.21–.72	Start your own business.	
	Conventional	10	.85	.11–.34	.38–.72	Keep inventory records.	
Big Five Inventory						I see myself as someone who..	(48)
	Openness to Experience	9	.78	.56–.81	.32–.58	… is clever; thinks a lot.	
	Conscientiousness	9	.87	.55–.82	.45–.71	… does things carefully and completely.	
	Extraversion	8	.86	.40–.72	.40–.73	… talks a lot.	
	Agreeableness	10	.79	.45–.90	.33–.58	… is kind and considerate to almost everyone.	
	Neuroticism	8	.76	.41–.71	.42–.55	… get nervous easily.	
Academic outcome	Satisfaction with the course contents (STUSAT-CC)	3	.85	.70–.81	.67–.79	I really enjoy what I'm studying.	(50)
	Satisfaction with the academic conditions (STUSAT-AC)	3	.74	.52–.61	.53–.60	Too little attention is paid to the needs of students at my university.	
	Satisfaction with coping with the academic load (STUSAT-LOAD)	3	.84	.59–.79	.68–.72	I often feel tired and exhausted from studying.	
	Intention to terminate studies or switch majors	5	.82	.07–.26	.54–.72	The thought often crosses my mind that my current course of studies is not for me.	(51)

Total sample size is *N* = 173; *k* = number of items; ω = reliability (42); *p_i_* = item difficulty; *r_it_* = item discrimination index.

Participants were recruited by program directors, who distributed the survey through email lists. In parallel, lecturers in some courses gave students time to fill out the survey. All students received information about data protection, study purpose, and procedures within the questionnaire. Participation in the survey was voluntary and no incentives were given. Informed consent was obtained from all participants before starting answering the questions. The ethics committee of TU Dortmund approved the project under the number “GEWK_2025-14”.

### 3.2 Measures

Overall, McDonald's omega is used to estimate internal consistency (42), with values of *ω* ≥ 0.70 considered adequate (43). All psychometric scales use items with a 5-point Likert scale ranging from 1 (=strongly disagree) to 5 (=strongly agree). Item difficulty is computed by Szota et al. (44) and is seen as the proportion of respondents endorsing higher response categories or in more technical: “item difficulties were calculated by dividing the sum of all participants’ scores on an item by the maximum possible score, resulting in *p_i_* between 0 and 1”.

#### 3.2.1 Vocational interests

The measurement instrument “The German O*NET Interest Profiler Short Form” by Roemer et al. (2), which was developed based on the work of Rounds et al. (45, 46) for the U.S., is used to assess vocational interests. Based on Holland's framework (1), vocational interests are described by the six components of the RIASEC model. Acceptable reliability is analyzed for Realistic (R; *ω* = .87; ten items; item sample: Lay brick or tile.), Investigative (I; *ω* = .90; ten items; item sample: Develop a new medicine.), Artistic (A; *ω* = .88; ten items; item sample: Compose or arrange music.), Social (S; *ω* = .80; ten items; item sample: Help conduct a group therapy session.), Enterprising (E; *ω* = .84; ten items; item sample: Start your own business.) and Conventional (C; *ω* = .85; ten items; item sample: Keep inventory records.). Table 1 presents the floor effects in the item difficulties for the scales Realistic (*p_i_* = .09), Conventional (*p_i_* = .11) and Enterprising (*p_i_* = .17). Item discrimination is seen as problematic for one item in the dimension Enterprising (*r_it_* = .21). Floor effects happen when many scores are at the lowest level, hiding real differences. Ceiling effects are also present in our sample for the Social dimension (*p_i_* = .81) that appear when many scores are at the highest level, also hiding differences. Both effects reduce data accuracy and make it hard to detect change or group differences (47).

#### 3.2.2 Personality traits

For the measurement of the Big Five personality traits, the instrument by Rammstedt and John (48) and originally by John et al. (49) is used. Data are collected for Openness to Experience (*ω* = .78; nine items; item sample: I see myself as someone who is clever; thinks a lot.), Conscientiousness (*ω* = .87; nine items; item sample: I see myself as someone who does things carefully and completely.), Extraversion (*ω* = .86; eight items; item sample: I see myself as someone who talks a lot.), Agreeableness (*ω* = .79; ten items; item sample: I see myself as someone who is kind and considerate to almost everyone.), and Neuroticism (*ω* = .76; eight items; item sample: I see myself as someone who get nervous easily.). In the Openness to Experience dimension, the item from the original inventory “I see myself as someone who likes work that is the same every time” is excluded due to its low item discrimination index (*r_it_* = –.01). This item shows a low item discrimination index in the study of Rammstedt and John (48), too. Reliability is considered tolerable. Table 1 shows a ceiling effect in item difficulties for the Agreeableness scale (*p_i_* = .90).

#### 3.2.3 Academic outcome

We divide academic outcome into the two components: study satisfaction and quitting the study program. Study satisfaction is measured using the three scales (1): satisfaction with course contents (STUSAT-CC; *ω* = .85; three items; item sample: I really enjoy what I'm studying.) (2), satisfaction with academic conditions (STUSAT-AC; *ω* = .74; three items; item sample: Too little attention is paid to the needs of students at my university.), and (3) satisfaction with coping with the academic load (STUSAT-LOAD; *ω* = .84; three items; item sample: I often feel tired and exhausted from studying.) developed by Westermann et al. (50). Intention to quit a study program is measured with the scale intention to terminate studies or switch majors (*ω* = .82; five items; item sample: The thought often crosses my mind that my current course of studies is not for me.) by Dresel and Grassinger (51). All reliabilities are considered acceptable. The intention to terminate studies or switch majors scale shows a floor effect, with the lowest item difficulty of *p_i_* = .07 (see Table 1).

#### 3.2.4 Congruence-index in vocational interests

There are numerous ways to evaluate vocational interest congruence, such as the C index or Iachan's M index (17, 52). Among the available congruence indices, Euclidean distance and Angular agreement were selected because they represent complementary approaches to person–environment (P–E) fit (17). Using both indices improves robustness, as different operationalizations of fit can produce different effect sizes and conclusions (53, 54). The combined use of distance- and direction-based indices increases construct coverage and follows recommendations in person–environment fit research to triangulate congruence rather than rely on a single measure. In a more detail view (17, 52), the Angular agreement assesses congruence by calculating the cosine of the angle between person and environment vectors in Holland's hexagonal model. It emphasizes directional similarity, where profiles pointing in similar directions yield high values close to 1, regardless of magnitude differences. This approach captures the circular structure of RIASEC types effectively, prioritizing ordinal proximity over absolute scores. Euclidean distance measures the straight-line separation between two RIASEC profile points in multidimensional space, computed as the square root of summed squared differences across dimensions. Lower distances signal higher congruence, as nearby profiles imply aligned interests and environments. It treats all dimensions equally. In short, the Angular agreement quantifies congruence as the angle between interest and occupational vectors, with 0 indicating no congruence and 1 indicating perfect congruence (17). In contrast, Euclidean distance quantifies incongruence by the distance between interest and occupational profile locations as well as a starting point 0 for congruence and end point is scale depend with 9.80 in our case (17). An advantage of Euclidean distance and Angular agreement is that they include all dimensional and individual measures of the RIASEC inventory. Another advantage is the use of the mean difference when estimating Euclidean distance and Angular agreement (17, 55).

In other words, Euclidean distance is calculated by summing the squared differences between the six person scores and the environment scores (RIASEC) and then taking the square root. The environmental scores follow the average of the constructs means from Table 2. Euclidean distance reflects the absolute discrepancy between profiles. Angular agreement was computed using the cosine of the angle between profiles. In a first step, the dot product is calculated and then divided by the product of their vector norms. The resulting value indicates similarity in profile direction independent of magnitude. As an example, see the “RealisticEnvironmentScore” in the SPSS-Syntax in the Supplementary Material. For the Realistic interest domain the mean value is 1.68. The syntaxes presented are intended to ensure reproducibility.

**Table 2:** Descriptive statistics, internal consistency estimates, and intercorrelations (*r*).

Interest domain	*M*	*SD*	ω	skewness	kurtosis	1.	2.	3.	4.	5.	6.
1. Realistic	1.68	0.70	.87	1.76	4.21	–					
2. Investigative	2.31	0.92	.90	0.53	−0.38	.53***	–				
3. Artistic	2.55	0.94	.88	0.15	−0.94	.31***	.35***	–			
4. Social	3.47	0.76	.80	−0.99	1.12	–.01	.16*	.30***	–		
5. Enterprising	2.12	0.76	.84	0.81	0.76	.43***	.42***	.32***	.14	–	
6. Conventional	1.72	0.67	.85	1.66	4.12	.71***	.47***	.35***	.01	.53***	–

Sample size is *N* = 173. Items are answered on a scale from 1 (=strongly disagree) to 5 (=strongly agree).

* *p* < .05.

** *p* < .01.

*** *p* < .001.

### 3.3 Data analyses and missing values

In our study each analysis is explicitly assigned to one study goal. Specifically, structural analyses test the theoretical geometry of the RIASEC model (primary goal). Analyses of the correlations with personality traits test the nomological position (secondary goal). Congruence analyses test whether distances between profiles relate to outcome variables (linking primary and secondary goal).

The analyses are conducted using IBM SPSS Statistics [version 29; (56)], unless otherwise specified. Skewness values outside the range of −1 to +1 are considered problematic for a normal distribution (57) and it is discussed that the results of a significance test could be biased, if a violation exists (58). We consider *p* < .05 as statistically significant (two-tailed).

In a first step, we analyze structural validity based on content validity by theoretical relevance, reliability through internal consistency by coefficient *ω* and item analyses using acceptable item difficulty between *p_i_* = .20 to .80 (59) and item discrimination *r_it_* ≥ .30 (60). Structural validity is further explored by determining intercorrelations and multidimensional scaling [MDS; (61)]. MDS helps visualize relationships between variables in a geometric space. By mapping data points, MDS can show whether their arrangement follows a hexagonal structure. This allows researchers to test if the hexagon model's geometric assumption fits the observed data. Although this is typically considered the standard method for such a procedure, a factor analysis is not estimated due to the relatively small sample size of 173 participants and the large number of 60 items due to a subject-to-item ratio of 2.9:1 in our case [at least *N* = 200 participants and ratios from 4 to 1 are recommended; (62)]. The software R Core Team (63) version 4.3.2 and package smacof version 2.1.7 (64), as well as the package tidyverse version 2.0.0 (65) are used. MDS visualizes the relations between the RIASEC domains in a two-dimensional space, where a hexagonal figure is awaited. Specifically, the correlation matrix is first converted into a distance matrix, followed by an interval MDS. The stress index in MDS shows how well the distances in the model match the original data (66). A lower stress value means a better fit. As a rule of thumb, stress values below 0.1 are very good, between 0.1 and 0.2 are fair, and above 0.2 indicate a poor fit. See Supplementary Material for the syntax. In MDS the Restricted Squared Correlation (RSQ) is a goodness-of-fit measure that is the proportion of variance in the observed dissimilarities. It is accounted for the distances in the low-dimensional configuration, where higher values (closer to 1) reflect a better representation of the data structure and lower values indicate a poorer fit.

Discriminant validity is examined by analyzing the relations between vocational interests and the Big Five Inventory using Pearson correlations (*r*). We consider *r* < .85 as an indicator for discriminant validity (67). Pearson's *r* is interpreted according to (68), who interprets values from *r* = |0.10| to *r* = |0.29| as small, *r* = |0.30| to *r* = |0.49| as medium, and *r* ≥ |0.50| as large. Evidence for criterion-related validity is assessed through associations between vocational interest congruence and academic outcome using Pearson correlations (*r*), with higher values of *r* indicating stronger evidence for criterion validity. If a correlation of *r* < |.10| is analyzed in one association between congruence index and outcome, then we see this as an indicator for non-criterion validity as well as non-significant results. Parallel, we estimate correlations according to Spearman (*r_s_*) to check robustness. Angular agreement and Euclidean distance indices are applied to analyze different aspects of congruence, like pattern similarity vs. absolute discrepancies. Using both indices allows a more comprehensive assessment rather than relying on a single operationalization. See the measures section for details. Different findings in the correlations *r* in the indices are seen as evidence that conclusions about criterion-related validity depend on the operationalization of congruence, rather than stating one index is better than the other.

The dataset of the sample of 173 participants contains missing values. The item non-response ranges between 0% and 17.3% (*M* = 5.32; *SD* = 5.59). In 88.48% of the measures and 142 cases (82.08% of the sample), no missing values are present. The Missing Values Analysis according to Littles' (69) test of Missing Completely at Random (MCAR) is not significant (*χ*^2^ = 860.58, *df* = 809, *p* = .102), indicating that the data is MCAR. In subsequent analyses, missing data are imputed using the method of multiple imputation by chained equations with the R package “mice” version 3.16.0, performing 100 imputations with predictive mean matching (PMM) across 50 iterations (m = 100, maxit = 50, seed = 500), yielding a completed dataset (70, 71).

A *post-hoc* and sensitivity power analysis is applied. We use the package pwr version 1.3.0 in R (72). See syntax in Supplementary Material. In line with the framework outlined by Faul et al. (73), we evaluated the statistical power for detecting Pearson correlations under our sample size of *N* = 173 and with a Bonferroni correction of 15 tests in terms of the six dimensions of pairwise comparison in RIASEC. The *post-hoc* power analysis indicated that for a medium effect size (*r* = .30) at a level of *α* = .05/15 (two-tailed) the statistical power is above.86. This result suggests that the present sample size was more than sufficient for detecting effects of this magnitude. Additionally, a sensitivity analysis showed that with *N* = 173 and *α* = .05/15, the minimum detectable effect size at 80% power is approximately *r* = .28. These results provide *post-hoc* justification that the sample size was adequate for the analytical strategy employed. Additional analyses and robustness checks are reported in the Supplementary Material.

## 4 Results

### 4.1 Descriptive statistics

In Table 2, the *M*, *SD*, and internal consistency estimates of the scale scores are reported. In the sample, the interest domains *Realistic* (*M* = 1.68; *SD* = 0.70) and *Conventional* (*M* = 1.72; *SD* = 0.67) have the lowest scores, whereas *Social* (*M* = 3.47; *SD* = 0.76) has the highest score. Internal consistency estimates are *ω* ≥ .80 for all interest domains. Normal distribution is considered problematic for the vocational interest domains *Realistic* with a skewness of 1.76, and *Conventional* with a skewness of 1.66, as they fall outside the range of −1 to +1.

### 4.2 Construct-related validity

#### 4.2.1 Structural validity

The correlations are analyzed based on the expected distance relations between RIASEC dimensions. Adjacent domains should have smaller distances (higher correlations) and opposite domains should show larger distances (lower correlations).

To answer RQ 1 regarding structural validity, we use content validity based on theoretical explanation and the empirical analyses of the intercorrelations (*r*), which are reported In Table 2. The correlation pattern shows only partial support for the RIASEC circumplex model. Expected adjacent relations appear, such as Realistic–Investigative (*r* = .53), Investigative–Artistic (*r* = .35), and Enterprising–Conventional (*r* = .53). A particularly strong link between Realistic and Conventional (*r* = .71) further suggests a structured interest space. However, non-adjacent correlations such as Investigative–Enterprising (*r* = .42) and Investigative–Conventional (*r* = .47) contradict the hexagon structure. The dimension Artistic also shows low specificity, correlating with the dimensions Realistic (*r* = .31), Social (*r* = .30), Conventional (*r* = .35), and Enterprising (*r* = .32). The positive and significant correlations of the dimension Social needs to be discussed for clarification. This result does not indicate a lack of construct validity. Moreover, this pattern could be seen as driven by a general positive manifold across interest domains. This could be explained by the fact that it overlaps conceptually with several other domains (particularly the dimensions Artistic and Enterprising). Overall, the circumplex structure is only partly confirmed and the results show that distances between dimensions do not fulfill the exact theoretical ordering, which is important for interpreting congruence measures in following analyses.

We further evaluated structural validity by item difficulties and item discrimination. We observe indications of floor effects in the scales Realistic for seven items, Conventional for seven items and Enterprising for three items (see also Table 2). Item discrimination is seen as problematic for one item in the dimension Enterprising (*r_it_* = .21). The kurtosis is above four in two scales that indicates a violation of normal distribution (see Table 2).

**Figure 1:**
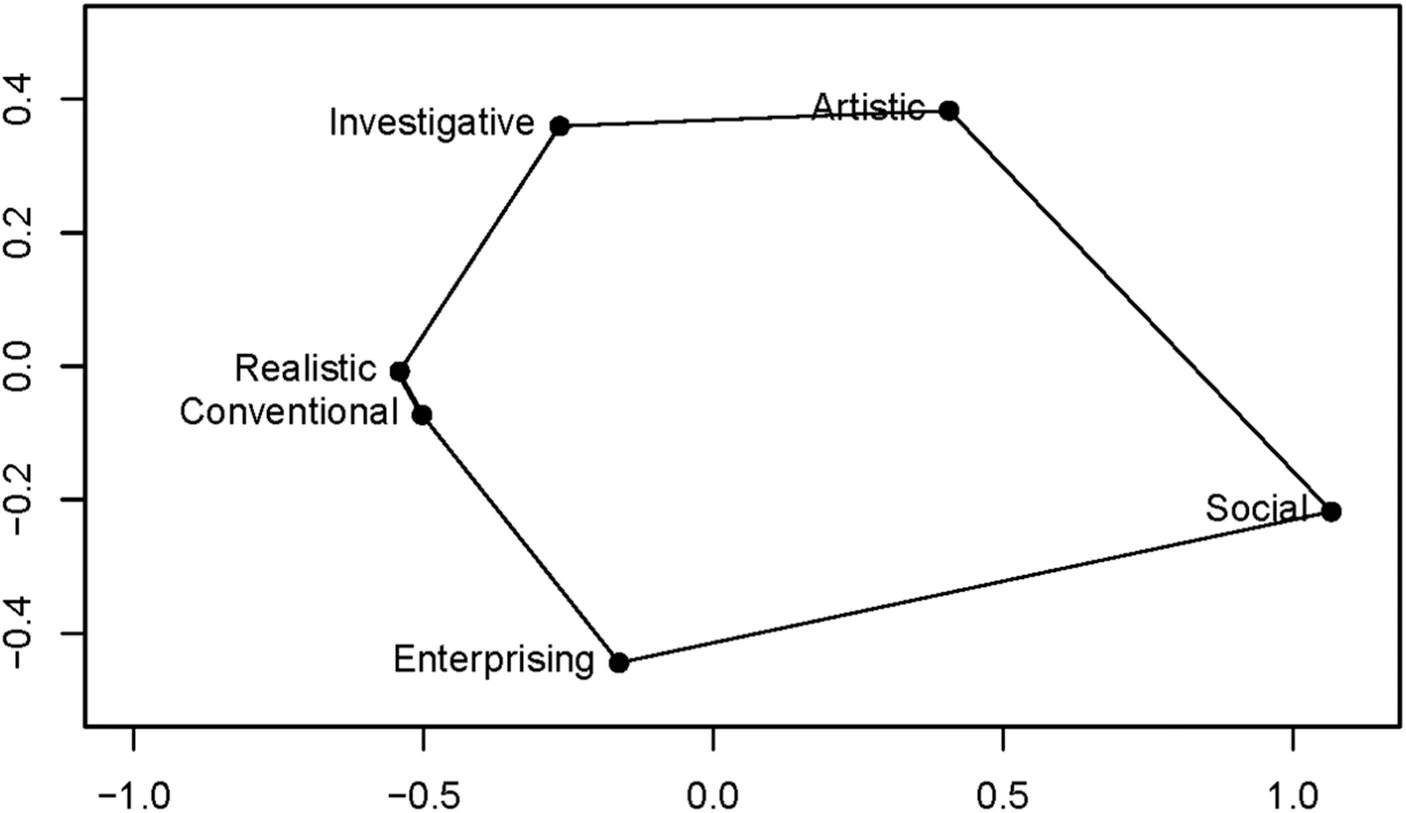
Metric multidimensional scaling solutions for the RIASEC scales. Note. Sample size is *N* = 173.

In the next step, the hexagonal structure is evaluated using MDS. A two-dimensional interval (metric) multidimensional scaling solution for the six objects shows a high goodness-of-fit with a RSQ value of .94 seeing as explained variance and indicating that the spatial configuration provides an excellent representation of the observed dissimilarities. Stress values range between .09 and .30 for the six dimensions, with .11 for the overall model. Figure 1 presents the visualized solution. The six vocational interest domains are ordered as awaited based on theoretical assumptions. In the estimated solution, the dimension Social is positioned far away from other vocational interests. In contrast, the dimension Conventional is located close to Realistic. The results of MDS address the secondary goal through visualizing distances between dimensions. The analyzed configuration allows an analysis if congruence indices based on geometric assumptions are theoretically justified in this empirical sample. Further robustness analyses are provided in the Supplementary Material.

#### 4.2.2 Discriminant validity

For testing hypothesis 1, we assess the instrumentś discriminant validity. Correlations between the six dimensions of vocational interest and the Big Five personality scale scores are estimated (see Table 3). As seen in previous research, moderate correlations are observed between Agreeableness-Social (*r* = .44) and Openness to Experience-Artistic (*r* = .42), as well as a low correlation between Extraversion-Social (*r* = .20). As awaited, negative correlations with small effect sizes are found for Agreeableness-Realistic (*r* = –.22), Agreeableness-Enterprising (*r* = –.23), Agreeableness-Conventional (*r* = –.21) and Neuroticism-Social [*r* = –.22; e.g., Roemer et al. (2)]. In contrast to previous studies, very small correlations are observed between Openness to Experience-Investigative (*r* = .06), and Enterprising-Extraversion (*r* = .05). The correlations are consistently small to medium in size and are in line with discriminant validity of the instrument (*r* < .85). This suggests that personality traits and vocational interests are related yet distinct constructs.

**Table 3:** Correlations between vocational interest scores and Big five personality trait scores.

Personality traits	Realistic	Investigative	Artistic	Social	Enterprising	Conventional
Openness to Experience	.03	.06	.42***	.19*	.09	.06
Conscientiousness	–.12	–.09	–.07	.22**	–.03	–.05
Extraversion	–.13	–.15	–.15	.20**	.05	–.19*
Agreeableness	–.22**	−17*	.01	.44***	–.23**	–.21**
Neuroticism	–.02	.00	–.03	–.22**	–.01	–.03

Sample size is *N* = 173.

* *p* < .05.

** *p* < .01.

*** *p* < .001.

### 4.3 Criterion-related validity

**Figure 2:**
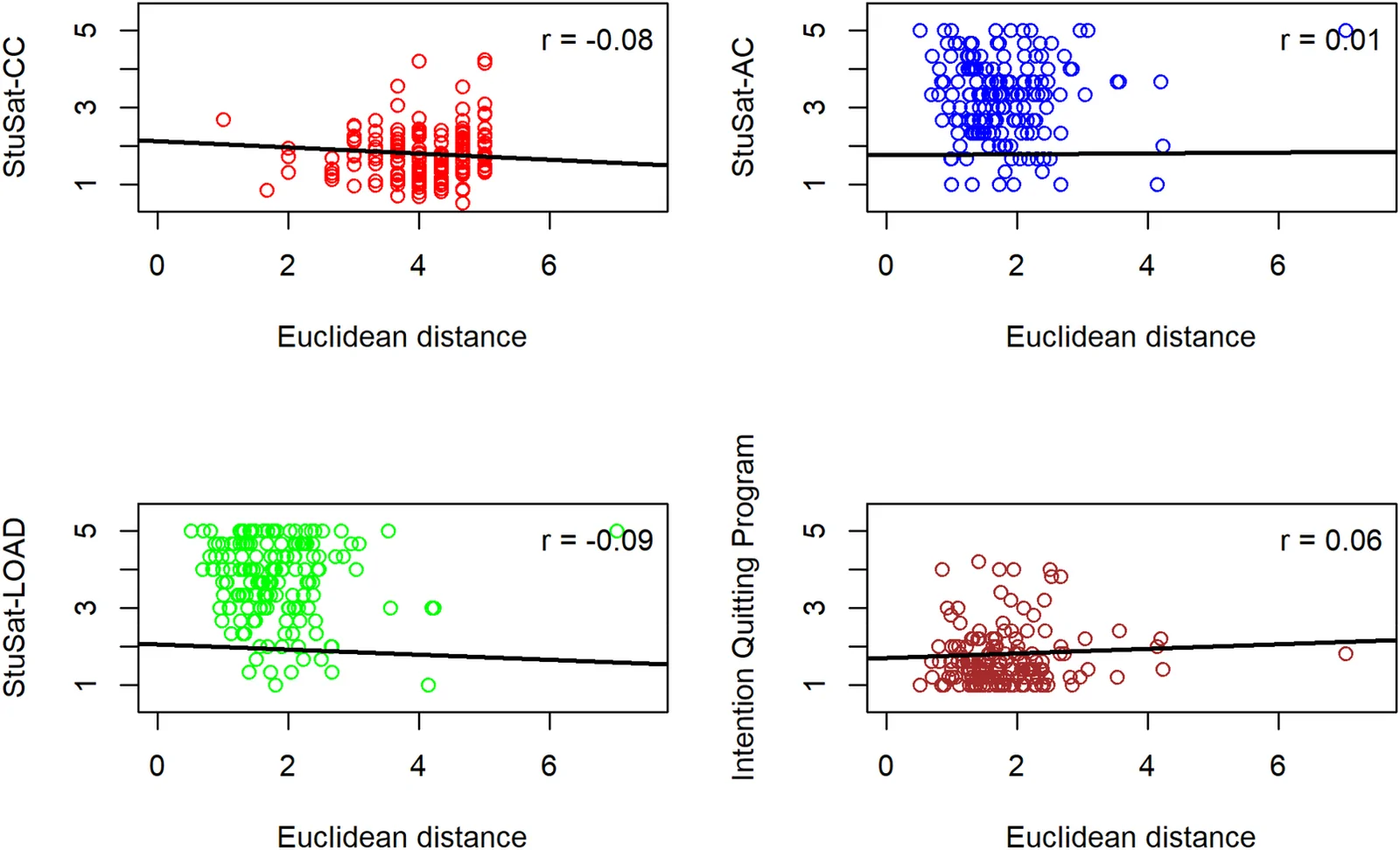
Scatterplots for congruence-Index Euclidean distance and academic outcome. Sample size is *N* = 173. Satisfaction with the course contents (STUSAT-CC); satisfaction with the academic conditions (STUSAT-AC); satisfaction with coping with the academic load (STUSAT-LOAD). For the Euclidean distance, smaller values indicate better congruence, with the starting point 0 for perfect congruence. The black line represents the regression line/line of best fit.

**Figure 3:**
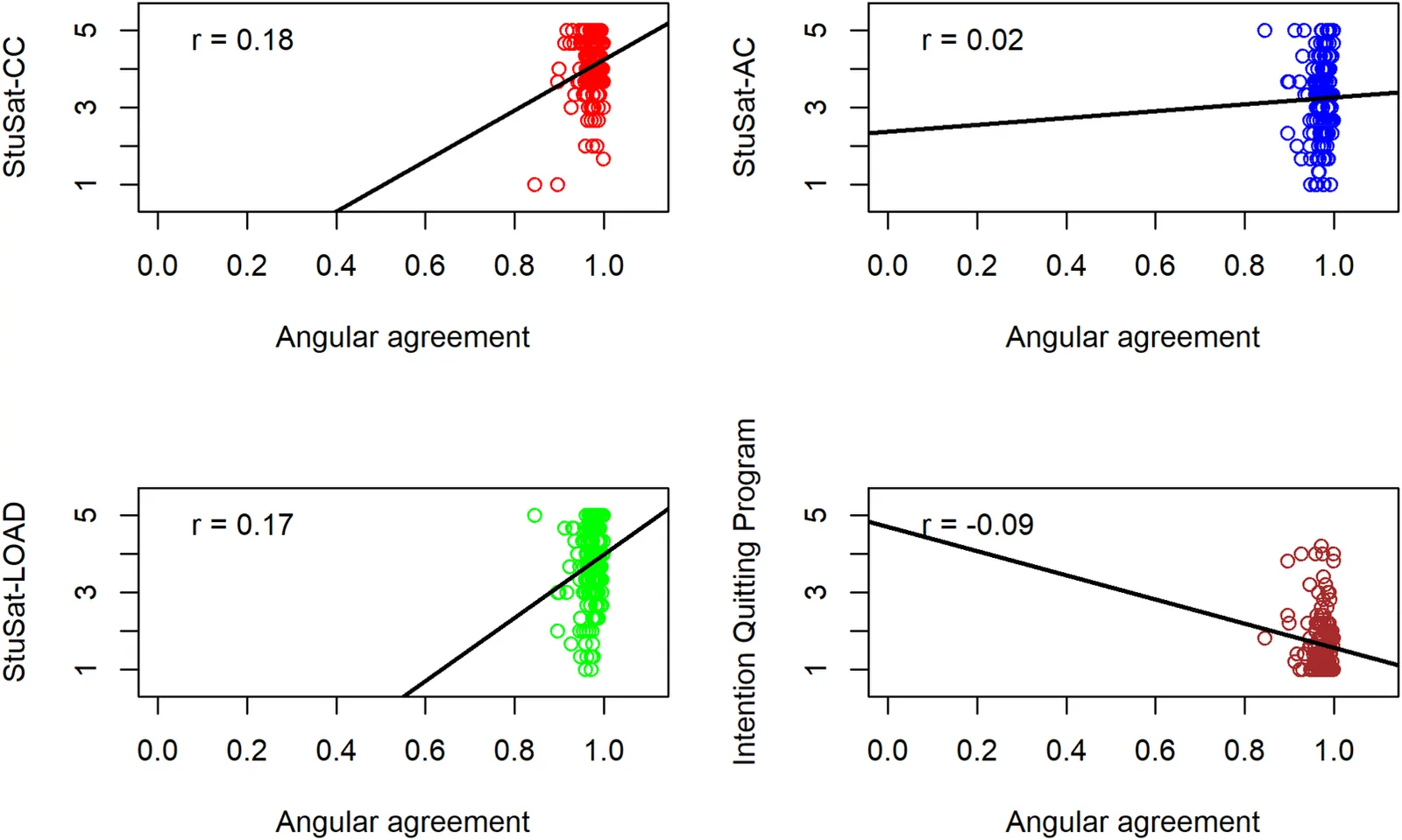
Scatterplots for congruence-Index angular agreement and academic outcome. Sample size is *N* = 173. Satisfaction with the course contents (STUSAT-CC); satisfaction with the academic conditions (STUSAT-AC); satisfaction with coping with the academic load (STUSAT-LOAD). For the Angular agreement, higher values indicate better congruence. Values of Angular agreement range between 0 and 1. The black line is the regression line/line of best fit.

The analyses in this chapter show if theoretically derived distances between person and environment profiles translate into meaningful outcome associations. To assess criterion-related validity for testing Hypothesis 2, correlations between congruent vocational interests and academic outcome are determined. Results are presented in Table 4 and visualized in Figures 2, 3.

**Table 4:** Correlations between vocational interest congruence scores and academic outcome.

Study variables	Euclidean distance		Angular agreement	
	*r*	*r_s_*	*r*	*r_s_*
STUSAT-CC	–.08	.07	.18*	.01
STUSAT-AC	.01	–.03	.02	.11
STUSAT-LOAD	–.09	–.09	.17*	.22**
Intention quitting study program	.06	.00	–.09	.01

Sample size is *N* = 173. Correlations labeled *r* refer to Pearson's correlation coefficient and *r_s_* indicates Spearman's rank correlation. Satisfaction with the course contents (STUSAT-CC); satisfaction with the academic conditions (STUSAT-AC); satisfaction with coping with the academic load (STUSAT-LOAD).

For the Euclidean distance, smaller values indicate better congruence, whereas for the Angular agreement, higher values indicate better congruence. Consequently, the mathematic signs of the correlations with other constructs are reversed for the Euclidean distance and the Angular agreement, but indicate the same.

* *p* < .05.

** *p* < .01.

*** *p* < .001.

First, we apply the Euclidean distance congruence-index, which ranges from 0 to higher positive values indicating less congruence. Negative correlations therefore indicate positive associations. Correlations show the awaited direction for three of the four academic outcome variables. There are correlations between Euclidean distance congruence-index and STUSAT-CC (*r* = –.08), STUSAT-LOAD (*r* = –.09), and quitting the study program (*r* = .06). These results are not statistically significant and so our assumption of criterion-related validity is not supported.

Second, we apply the Angular agreement congruence-index, which ranges from 0 (no congruence of vocational interest) to 1 (full congruence). We observe very small but statistically significant associations between the Angular agreement congruence-index and STUSAT-CC (*r* = .18) and STUSAT-LOAD (*r* = .17).

The different results from the two indices of Euclidean distance and Angular agreement suggest that the operationalization of profile distance influences the observed associations, highlighting measurement-dependent variability in criterion validity. These analyses provide important evidence for the empirical findings related to the secondary goal of exploring dimensional distances.

In the analyses of similarities between correlations according to Pearson's (*r*) and Spearman's (*r_s_*) in Table 4 it is seen that coefficients are consistent across most conditions in sign and generally indicate weak relations. Inspecting congruence of Euclidean distance, correlations are near zero with no indicating only very weak linear and monotonic associations. Minor differences exist, for example with STUSAT-CC that may reflect distributional characteristics, although no systematic non-linearity can be inferred from the present data. Focusing angular agreement, STUSAT-LOAD shows consistent positive and significant effects across both coefficients, indicating a weak but stable monotonic relationship. In contrast, STUSAT-CC indicates a significant Pearson but non-significant Spearman correlation, suggesting that the association may be sensitive to distributional characteristics, although this interpretation remains tentative. After all, the analyzed differences emphasize minor distributional influences rather than substantive contradictions between correlation approaches.

Overall, the results provide limited and inconsistent evidence for criterion-related validity. Effect sizes remain small (all *r* < .20) and show limited practical relevance despite occasional statistical significance. As a consequence, hypothesis 2 is not confirmed. These findings suggest that criterion-related validity may be test-dependent and should be interpreted with caution depending on how congruence is operationalized. Sensitivity analyses are reported in the Supplementary Material.

## 5 Discussion

### 5.1 Summary

The results are analyzed in light of the following study goals. The first goal was to test if theoretical assumptions of the RIASEC model are adequately replicated in the data. Second, we tested how the analyzed distances between dimensions influence conclusions about congruence.

We examine the psychometric quality of a vocational interest measurement instrument, the German O*NET Interest Profiler Short Form by Roemer et al. (2), which is based on the RIASEC theory (1). Our focus is on structural validity (RQ 1), discriminant validity (Hypothesis 1), and criterion-related validity (Hypothesis 2). Analyses are based on data from an understudied population, students enrolled in rehabilitation education at German universities, to test the robustness of the instrument, as most vocational interest measurement instruments are developed and tested in the U.S (12). We also contribute to the analysis of the effects of different congruence interest indices on the results (14, 17).

The empirical results on structural validity provide first evidence supporting the assumptions (RQ 1) in our data. The subscales consistently showed high internal consistency and satisfactory item discrimination. The analyzed correlations show that the results are generally in line with the expectation that adjacent interest domains show stronger relationships than more distant or opposite ones, as seen, for example between the correlations of Realistic–Investigative and Enterprising–Conventional. Parallel, some non-adjacent correlations are also relatively high, like Investigative–Enterprising and Investigative–Conventional, which weakens a clear circumplex pattern. Overall, the results only partially support the hexagonal structure of interest domains proposed by Holland (1). Additional analyses using MDS reveal an ordered arrangement of the domains, consistent with theoretical assumptions. However, a moderate fit solution based on stress values indicates discrepancies between the distances in the original data and those in the two-dimensional solution. The calculated solution shows that the position of the Social interest is positioned far from the other vocational interests, suggesting that this domain has unique properties that make it very different from most of the domains. In contrast, the Conventional vocational interest is located close to Realistic, indicating a very high similarity among survey participants. Consequently, the hexagonal structure represents an idealized model of the vocational interest relations [see the assumption of the calculus hypothesis; Leon et al. (13)], while empirical research indicates a “misshapen polygon” (1) solution.

In summary, the results provide evidence for the postulated hexagonal structure of vocational interest proposed by Holland (1), which support the structural validity of the instrument in this population. However, it must be emphasized that some items show floor effects that indicate small variance in lower trait ranges. Two scales showed problematic deviations from a normal distribution, which should be kept in mind when interpreting statistical models. To address the potential impact of these distributional issues, all analyses are done in parallel using log-transformed scores from the six dimensions for checking the robustness for skewness effects. Importantly, the correlation between Realistic and Conventional remained high after log transformation, decreasing only from *r* = .71 to *r* = .67. This fact suggests that floor effects alone are unlikely to account for the observed association.

The resulting correlation matrix and MDS remain almost unchanged and indicate that the observed structure is robust against the identified floor effects. Overall, the evidence suggests a generally stable and theoretically based factor structure, indicating that the structural validity of the instrument could be supported. It is considered important that deviations from the expected geometric ordering indicate that distances between RIASEC dimensions are not equal. This finding directly limits how congruence indices based on these distances can be interpreted.

The associations between Realistic and Conventional is notably higher in our study than in meta-analyses (e.g., 31) and prior validation research (2). Consequently, the presented result suggests that the separation between these domains is reduced in the present sample. One possible reason could be restricted variance, as both domains show comparatively low mean levels and limited dispersion, consistent with a potential floor effect. Parallel, the special educational setting could contribute to a fusion of these domains. A possible reason is the fact that both realistic and conventional activities may be similarly unattractive to the students, thereby reducing the interest space. Empirical reasons could be seen in the explanations of restricted variance against context-specific fusion. Consequently, we conducted further analyses on both raw and log-transformed scores. Specifically, we examined attenuation patterns in the correlation structure, and evaluated whether variance restriction could plausibly account for the magnitude of the Realistic–Conventional correlation. It should be noted that although this large correlation affects the local structure in this pattern, it does not fully determine the overall pattern of associations across all the RIASEC dimensions. The results of further analyses indicate that log-transformation and variance adjustments only marginally reduce the Realistic and Conventional correlation and do not meaningfully restore variance to levels comparable with other RIASEC scales. This pattern suggests that restricted variance alone is insufficient to explain the observed association. To address this, we did a parallel MDS analysis in which the observed Realistic and Conventional correlation was replaced by the meta-analytic estimate (31) and that is done for the purpose of a stability check of the RIASEC structure. The result is presented in Figure 1 of the Supplementary Material. This analysis was conducted as a robustness check. The overall circular ordering of RIASEC types remain partially stable. There is consistent proximity between theoretically adjacent types (e.g., Realistic and Investigative as well as Artistic and Social). The previously observed small distance between Realistic and Conventional has increased. The social type no longer occupied a strongly peripheral position. However, the hexagonal structure is not fully recovered. Taken together, the convergence of (a) persistent correlation after log-transformation and (b) insufficient variance correction provides stronger support for a substantive, context-related interpretation (i.e., a partial fusion of Realistic and Conventional interests in rehabilitation education students) rather than a purely psychometric artifact due to floor effects. This indicates that the overall pattern is only partially sensitive to the substitution of the inflated Realistic and Conventional correlation. These findings suggest that the observed structure is not an artefact of the elevated Realistic and Conventional correlation alone. This correlation does contribute to deviations from the ideal RIASEC geometry.

For discriminant validity (Hypothesis 1), our findings indicate that the correlations between interest scores and Big Five personality traits generally align with previous research (2, 31). Most correlations are small, providing empirical support for viewing the Big Five personality traits and vocational interests as independent constructs. Therefore, RQ2 is supported. H1 is confirmed. Additionally, the results provide first evidence that the instrument scores are linked to the theoretically relevant personality constructs of the Big Five that is consistent with expectations derived from the nomological network of vocational interests. There are a few deviations from previous findings, such as the correlation between Openness to Experience and Investigative Interest, which may be explained by the culturally specific sample of students in rehabilitation education, like the predominance of female participants, or the location of the universities. Furthermore, the Social dimension in RIASEC correlates with all Big Five traits, because it reflects interpersonal orientation and people with social interests tend to be more open, conscientious, extraverted, agreeable, and less neurotic. This result could be seen in the context that this pattern is especially strong in a sample of rehabilitation education students, who are typically drawn to helping professions. Another view for this result could be that these correlations may be reinforced by the specific sample. It is possible that the findings likely reflect sample characteristics rather than general effects. In summary, these results provide initial evidence for the discriminant validity of the evaluated measurement instrument. These findings support that the instrument measures within the theoretical nomological network as expected.

Criterion-related validity should be interpreted in light of the observed distance structure between RIASEC dimensions and outcome variables. We examined criterion-related validity in Hypothesis 2 and followed the assumption that higher vocational interest congruence is positively associated with study program satisfaction and negatively associated with the intention to drop out of the study program, as suggested by research in vocational behavior (3, 6–8), and higher education (10). To assess this, we employed the two congruence indices: Euclidean distance and Angular agreement. Although most correlations were in the expected direction, none of the effects based on Euclidean distance and only two of the four effects in the index of angular agreement are statistically significant. Parallel, only two out of eight tests reached significance, specifically for study program satisfaction and vocational interest congruence. Overall, Hypothesis 2 is not supported. Criterion-related validity is weak in this sample. The very small effects between vocational interest congruence and satisfaction with academic conditions (STUSAT-AC) may result from other variables, such as academic self-concept, influencing the bivariate correlation (74). However, not all correlations are significant. Following Van Iddekinge et al. (8), who report a small effect size for the correlation between vocational interests and turnover intentions in their meta-analysis, we note that results from other industries cannot be directly transferred to research in higher education, as the effects in our study are smaller. Possible reasons include the fact that short-term and business aspects often play a greater role in working life than studying in higher education. In summary, the results of this study do not provide robust evidence for criterion-related validity and suggest that observed associations are sensitive to the operationalization of congruence. Vocational interest congruence was not consistently related to outcome variables. A reason could be the fact that the geometric structure is only partially supported, distances between profiles may not accurately reflect true psychological proximity. This could explain why congruence indices show weak and not consistent associations with outcome variables. As a consequence, the results show that limited structural validity and constrains in the interpretation of congruence effects exist. Results in our study should not only be interpreted as a psychometric limitation of the German O*NET Interest Profiler Short Form. Moreover, the results could show a theoretical boundary condition of Holland's congruence framework itself. Here it is assumed that the congruence assumption applies under educational or occupational environments and can be represented by sufficiently coherent and stable interest profiles. This idea is more plausible in highly standardized professions, like medicine, nursing, or school teaching, where educational pathways correspond relatively directly to clearly defined occupational environments [Leichner et al. (75)]. In contrast, rehabilitation education is characterized by heterogeneous, multidirectional, and comparatively diffuse professional trajectories and pathways. Students in rehabilitation education may enter working fields in educational, therapeutic, counselling, administrative, or social-support that differ substantially in their environmental characteristics. As a consequence, the environmental profile used for congruence estimation may not adequately capture the diversity of relevant environments. Under this structural conditions, even mathematically sophisticated congruence indices may possess limited explanatory power because the term “environment” itself is not enough specified. This interpretation is in line with recent meta-analytic evidence that shows congruence effects in higher education are generally smaller than in occupational contexts (10). Our findings extend this point of view in the literature by suggesting that program-specific environmental ambiguity may partly explain why criterion validity weakens in some academic settings. Overall, the present results are theoretically informative because they indicate that the predictive use of congruence indices may depend not only on psychometric quality or index selection, but also on the degree of occupational specificity inherent in the educational environment itself.

### 5.2 Practical implication and limitation

This measurement instrument has several practical implications for higher education and student counseling. First, it can support potential and current students in career guidance and study program selection through Online Self-Assessments [OSA; (76, 77)]. Based on the Person-Environment-Fit theory (27), these tools enhance the match between individual interests, skills, and study environments, thereby increasing student satisfaction and academic success (10). However, the findings of this study suggest that practical implications should be differentiated across congruence operationalizations. In the current sample, Angular agreement shows more consistent associations with academic outcomes than Euclidean distance. A possible explanation is that the Angular agreement index captures profile similarity independent of absolute score magnitude and is therefore less sensitive to deviations from the idealized RIASEC geometry observed in this study. On the other hand, the Euclidean distance index assumes more stable and metrically interpretable distances between RIASEC dimensions, an assumption that was only partially supported by the empirical structure. Consequently, for counselling contexts in heterogeneous educational environments such as rehabilitation education, Angular agreement may currently represent the more appropriate exploratory congruence indicator, whereas Euclidean distance should be interpreted with greater caution. It should be noted that the observed effect sizes remain small and do not justify high-stakes individual decision-making based solely on congruence scores. Secondly, aggregated assessment data from this instrument can help universities evaluate and align curricula with students' interests (78). This alignment helps identify whether courses support individual motivation, engagement, and career development. Therefore, integrating these results into quality management ensures that study programs remain relevant, student-centered, and responsive to labor market needs.

However, our study has several limitations. The sample size of 173 participants is relatively small, and the number of male respondents is low (fewer than 30 persons) due to the high proportion of female students in this program (79). This makes it difficult to compare genders and to examine test fairness through measurement invariance testing across different subgroups (80). Another limitation is the low reliability of satisfaction with the academic conditions (STUSAT-AC) with *ω* = .74. This may explain the weak correlations with the congruence indices of vocational interests. Moreover, as the study relies on cross-sectional data, longitudinal data would be preferable, as they allow estimating more rigorous analyses, such as test-retest reliability or predictive validity (81). In addition, floor and ceiling effects were observed for the Realistic and Conventional scales. This restricts the ability to detect differences between individuals, especially at extremely high and low scores (82). Normal distribution is violated in some case and as a consequence significant results could be biased (56). Because of the limited sample size, exploratory or confirmatory factor analyses could not be conducted and this limits the ability to evaluate the factorial validity of the measures in this sample. Moreover, most existing correlations between congruence and other variables are not significant, and the less significant ones are very small, suggesting that these associations have limited practical relevance in the present sample. It must be mended that the angular distance measure used to evaluate criterion validity is highly skewed, which could influence the robustness and interpretability of the observed relationships.

A further limitation in this study is the low response rate of approximately 15% that could entail nonresponse bias. However, as noted by Survey Methodology, response rates alone are not sufficient to assess the extent of bias and instead, potential differences between respondents and non-respondents are decisive (83). In terms of observable characteristics, the sample corresponds to the known population structure of rehabilitation education students at TU Dortmund, like gender distribution with approximately 85% female in WS 2022/23 and WS 2023/24, compared to 83% in the present sample (79). This reduces a few concerns about major demographic distortion, although selective participation related to study satisfaction or dropout intentions cannot be ruled out entirely.

Another limitation of this study is the unequal proportion of participations from universities in this sample, with TU Dortmund University accounting for more than half. This sample could raise concerns about institution-specific effects, like satisfaction outcomes. However, TU Dortmund University and Humboldt-Universität zu Berlin are indeed the leading universities in Germany with the highest number of students in the field of rehabilitation education. This fact is also reflected in our sample. Moreover, controlling for university affiliation in a multivariate analysis of variance (MANOVA) identical associations between Angular agreement and student satisfaction outcomes are determined. Thus, institutional affiliation appeared not as a confounding variable in the analysis.

### 5.3 Outlook

Based on our findings, new research questions arise. One challenge is analyzing whether interest in the rehabilitation research study program remains stable over time. Interest stability has been observed in other student programs in higher education (84). Another desirable approach is to apply item response theory to test this instrument. The number of ten items per dimension provides an argument for such analyses, since classical test theory relies on fewer items, compared to the item response analysis (85). Future researchers should also investigate construct validation in greater depth. One possible way to do this is by applying multitrait–multimethod (MTMM) designs (86).

The operationalization of the environmental profile is challenging in rehabilitation education because the study program does not correspond to a single occupational endpoint. This structural ambiguity limits the interpretability of congruence estimates independently of the mathematical properties of the indices themselves. Future research should therefore investigate whether Holland-based congruence models operate differently in educational contexts with diffuse vs. highly standardized occupational destinations.

Overall, the study suggests that validity evidence depends on the extent to which theoretical distance assumptions are met. It could be assumed that when the geometric structure deviates, then conclusions about congruence and outcomes get less robust.

## 6 Conclusion

The results of the presented study must be interpreted in relation to both study goals. Roemer et al. (2) proposed the German O*NET Interest Profiler Short Form. Following their call, we examined the robustness of its psychometric quality and applied the instrument to the understudied population of university students in rehabilitation education. Our findings provide initial evidence for structural validity, discriminant validity and weak evidence for criterion-related validity of this free, German-language instrument for assessing vocational interests according to Holland's (1) theory. Further research is needed to verify its robustness, for example by evaluating it in other populations or applying modified methods and advanced statistical techniques. However, the present findings must also be seen in context that the validity of congruence approaches may depend on the occupational specificity as well as on educational context. In study programs with diffuse and heterogeneous professional pathways, such as rehabilitation education, weak congruence effects may represent theoretically meaningful limits of person–environment fit models rather than merely methodological shortcomings. Following the study goals in this research the following research should focus on modelling distances between RIASEC dimensions in more detail, as these are central for both validating the structure and interpreting congruence effects.

## Data Availability

The raw data supporting the conclusions of this article will be made available by the authors, without undue reservation.
