# Supplementary material for: A preliminary analysis of the psychometric properties of the German O*NET interest profiler short form in rehabilitation education
Source: Front Rehabil Sci. 2026 Aug 20;7:1789335. doi: 10.3389/fresc.2026.1789335 (PMC13538876; doi:10.3389/fresc.2026.1789335)
Supplement: Supplementary file 1 [file Datasheet1.pdf]

Supplemental Material

This section contains additional analyses and robustness checks that support the main results of the study.

Figure 1

Robustness check of the multidimensional scaling

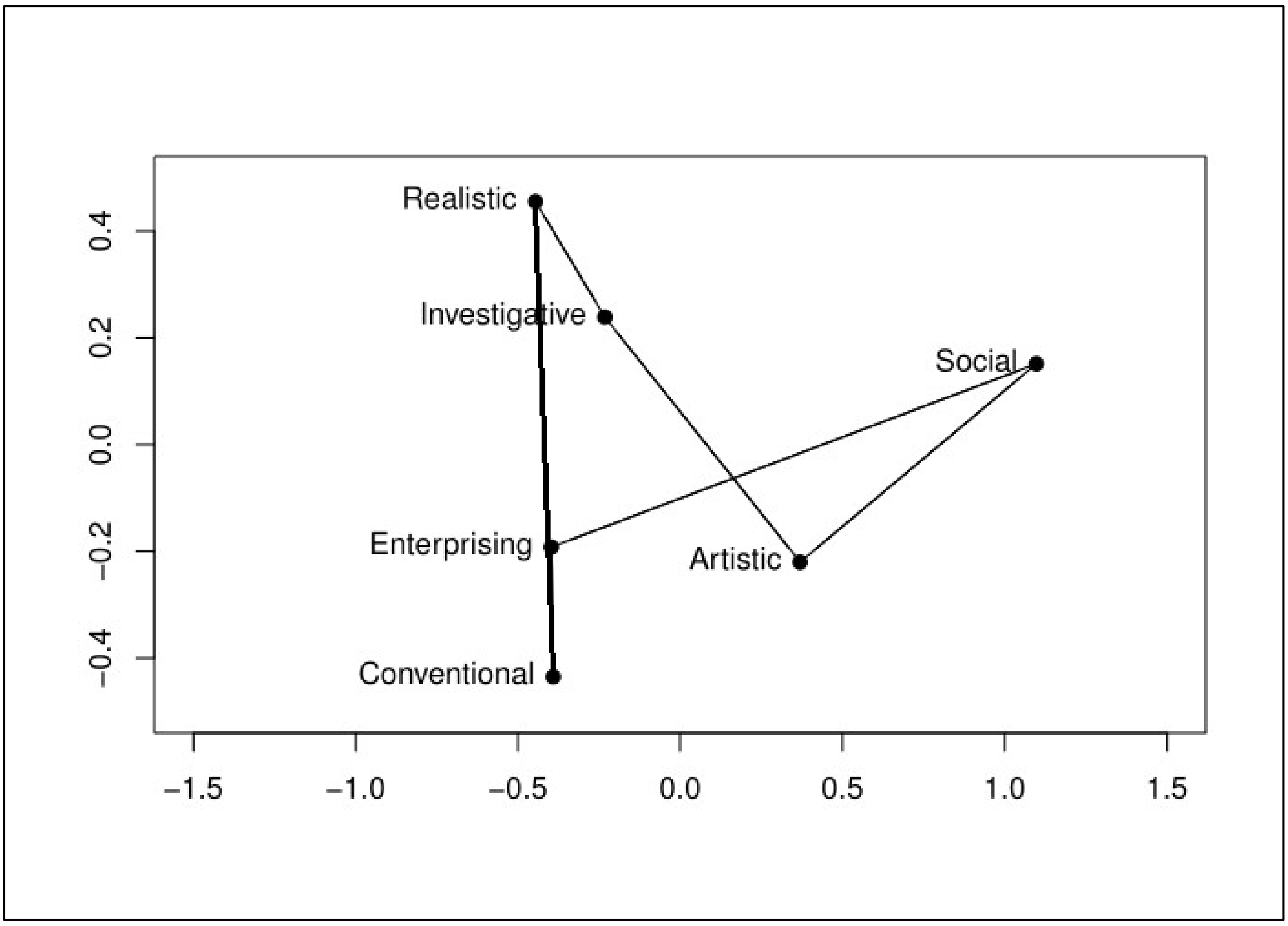

Note. Sample size is N = 173. Analysis use correlation from this study and the meta-analytic Realistic and Conventional correlation (ρ = .27, (31)).

Table 1

Sample characteristics

	Absolute numbers	Proportion/mean (*M*) with standard deviation (SD)
Age		*M* = 24.57 (*SD* = 5.29)
Gender		
Female	143	83%
Male	21	12%
Non-binary	9	5%
Study program		
Bachelor	126	73%
Master	47	27%
University		
TU Dortmund University	95	55%
Humboldt-Universität zu Berlin	50	29%
Carl von Ossietzky Universität	23	13%
Oldenburg		
University of Cologne	5	3%

Note. *N* = 173.

SPSS-Syntax for Euclidean distance for reproducibility

```
COMPUTE euclid_dist = SQRT(
  (RealisticPersonScore - RealisticEnvironmentScore)**2 +
  (InvestigativePersonScore - InvestigativeEnvironmentScore)**2 +
  (ArtisticPersonScore - ArtisticEnvironmentScore)**2 +
  (SocialPersonScore - SocialEnvironmentScore)**2 +
  (EnterprisingPersonScore - EnterprisingEnvironmentScore)**2 +
  (ConventionalPersonScore - ConventionalEnvironmentScore)**2
).
EXECUTE.
```

SPSS-Syntax for Angular agreement for reproducibility

```
COMPUTE dot_product = RealisticPersonScore*RealisticEnvironmentScore +
InvestigativePersonScore*InvestigativeEnvironmentScore +
ArtisticPersonScore*ArtisticEnvironmentScore + SocialPersonScore*SocialEnvironmentScore +
EnterprisingPersonScore*EnterprisingEnvironmentScore +
ConventionalPersonScore*ConventionalEnvironmentScore.

COMPUTE norm_pers = SQRT(RealisticPersonScore**2 + InvestigativePersonScore**2 +
ArtisticPersonScore**2 + SocialPersonScore**2 + EnterprisingPersonScore**2 +
ConventionalPersonScore**2).
COMPUTE norm_env = SQRT(RealisticEnvironmentScore**2 + InvestigativeEnvironmentScore**2 +
ArtisticEnvironmentScore**2 + SocialEnvironmentScore**2 + EnterprisingEnvionmentScore **2 +
ConventionalEnvironmentScore**2).

COMPUTE cos_theta = dot_product / (norm_pers * norm_env).
```

R-Syntax for multidimensional scaling for reproducibility

```r
library(smacof)

library(tidyverse)

dat <- matrix(c(
0.00000,0.48580,0.58822,0.71099,0.53292, 0.38341,
0.48580,0.00000,0.57053,0.64807,0.53991, 0.51720,
0.58822,0.57053,0.00000,0.59119,0.58395, 0.57184,
0.71099,0.64807,0.59119,0.00000,0.65422, 0.70214,
0.53292,0.53991,0.58395,0.65422,0.00000, 0.48425,
0.38341,0.51720,0.57184,0.70214,0.48425, 0.00000
), nrow=6, byrow=TRUE)
d <- as.dist(dat)

mdsfit <- smacofSym(d, ndim = 2, type = "interval",weightmat=NULL, init =
"torgerson",ties="primary",verbose=FALSE, relax = FALSE, modulus = 1, itmax = 500)

mdsfit  %>%  summary()
mdsfit$stress

fitted_dist <- dist(mdsfit$conf)

rsq <- cor(as.vector(d), as.vector(fitted_dist))^2

rsq
```

R-Syntax for post-hoc and sensitivity power analyses for reproducibility

```
library(pwr)

pwr.r.test(n = 173, r = 0.30, sig.level = 0.05/15)

pwr.r.test(n = 173, power = 0.80, sig.level = 0.05/15, alternative = "two.sided")
```
